# Bacterial LuxR solos have evolved to respond to different molecules including signals from plants

**DOI:** 10.3389/fpls.2013.00447

**Published:** 2013-11-12

**Authors:** Hitendra K. Patel, Zulma R. Suárez-Moreno, Giuliano Degrassi, Sujatha Subramoni, Juan F. González, Vittorio Venturi

**Affiliations:** International Centre for Genetic Engineering and BiotechnologyTrieste, Italy

**Keywords:** quorum sensing, *N*-acyl homoserine lactones, interkingdom signaling, LuxR solos, plant signals

## Abstract

A future challenge will be understanding the extensive communication that most likely takes place in bacterial interspecies and interkingdom signaling between plants and bacteria. A major bacterial inter-cellular signaling system in Gram-negative bacteria is LuxI/R quorum sensing (QS) based on the production (via the LuxI-family proteins) and detection (via the LuxR-family proteins) of *N*-acyl homoserine lactones (AHLs) signaling molecules. LuxR proteins which have the same modular structure of QS LuxRs but are devoid of a cognate LuxI AHL synthase are called solos. LuxR solos have been shown to be responsible to respond to exogenous AHLs produced by neighboring cells as well endogenously produced AHLs. It is now also evident that some LuxR proteins have evolved from the ability to binding AHLs and respond to other molecules/signals. For example, recent research has shown that a sub-family of LuxR solos responds to small molecules produced by plants. This indicates the presence of a uni-directional interkingdom signaling system occurring from plants to bacteria. In addition LuxR solos have now been also implicated to respond to endogenously produced signals which are not AHLs. In this Mini Review article we will discuss current trends and implications of the role of LuxR solos in bacterial responses to other signals using proteins related to AHL QS systems.

## BACTERIAL QUORUM SENSING SYSTEMS AND LuxR SOLOS

The most common bacterial quorum sensing (QS) system in Gram-negative bacteria occurs via *N*-acyl homoserine lactones (AHLs). A canonical QS system consists of a LuxI-family synthase responsible for synthesizing the AHL signal which then interacts at quorum concentrations with the cognate LuxR-family transcription factors affecting gene expression (Fuqua et al., 2001). Importantly, AHLs vary in their structure having different acyl chain lengths (from 4 to 20 carbons) with variation in the oxidation state at position C3 on the acyl chain (being a methylene, or a ketone, or can be hydroxylated).

Extensive research on bacterial AHL QS revealed that many plant associated bacteria (PAB) employ QS for the regulation of virulence associated functions of phyto-pathogens and of beneficial traits in plant-growth-promoting bacteria (Cha et al., 1998; Gonzalez and Marketon, 2003; Von Bodman et al., 2003; Danhorn and Fuqua, 2007). Researchers are also currently studying AHLs as interkingdom signals being perceived by plants. In fact, several studies are indicating that plants respond to bacterial AHLs regulating plant gene expression affecting important phenotypes (Mae et al., 2001; Mathesius et al., 2003; Toth et al., 2004; Schuhegger et al., 2006; You et al., 2006; von Rad et al., 2008; Schikora et al., 2011; Schenk et al., 2012). Importantly, plants also interfere with bacterial QS systems by producing low molecular weight compounds which mimic QS signals acting as agonists or antagonists to bacterial AHL QS systems (Teplitski et al., 2000; Manefield et al., 2002; Gao et al., 2003; Bauer and Mathesius, 2004; Teplitski et al., 2004; Keshavan et al., 2005; Degrassi et al., 2007). QS systems in PAB therefore appear to be influenced by plant molecules but also affect plant gene expression via AHLs.

Quorum sensing LuxR-family proteins are transcriptional regulators that bind AHLs being approximately 250 amino acids long and consisting of two domains separated by a short linker region; an autoinducer-binding domain is located in the *N*-terminal region (Shadel et al., 1990; Slock et al., 1990) and a DNA-binding helix-turn-helix (HTH) domain is positioned at the C-terminal region (Choi and Greenberg, 1991, 1992; Fuqua and Winans, 1994). LuxR-type proteins regulate transcription by binding to DNA in gene promoter regions at a conserved site called a *lux* box (Devine et al., 1989; Stevens and Greenberg, 1997). QS LuxRs display surprisingly low homologies (18–25%); 95% however, share nine highly conserved amino acid residues (Whitehead et al., 2001; Zhang et al., 2002). Six of these are hydrophobic or aromatic and form the cavity of the AHL-binding domain and the remaining three are in the HTH domain (Fuqua et al., 1996). AHL QS systems consist of the *luxI/R* genes, which are almost always located genetically adjacent to each other. Sequencing bacterial genomes showed that proteobacteria possess a family of proteins highly similar to QS LuxRs which do not possess a cognate LuxI protein associated with them; these have been called orphans and solos (Fuqua, 2006; Subramoni and Venturi, 2009).

LuxR solos consist of the same modular structure having an AHL binding domain in the *N*-terminus and a DNA binding HTH at the C-terminus. Some have been shown to expand the regulatory targets of the canonical complete AHL QS systems by responding to endogenous or exogenous AHLs. In the latter case, they regulate target genes by “eaves-dropping” on exogenously provided AHL signals produced by neighboring bacteria (Ahmer, 2004). Two well studied such LuxR solos are QscR from* P. aeruginosa* which responds to endogenously produced AHLs (Chugani et al., 2001; Lequette et al., 2006) and SdiA of *Salmonella enterica* and *Escherichia coli* which eavesdrop on AHLs produced by neighboring bacteria (Ahmer et al., 1998; Michael et al., 2001; Ahmer, 2004; Yao et al., 2006). A sub-family of these LuxR solos only found in PAB have lost the capacity to bind AHLs and instead evolved the ability to respond to low-molecular weight plant compounds. This uni-directional interkingdom signaling circuit has therefore evolved from canonical AHL QS systems where the LuxR protein no longer responds to endogenously produced AHLs, but to plant signals (Gonzalez and Venturi, 2013; Venturi and Fuqua, 2013). In addition LuxR solos have also recently been shown to respond to endogenous signals which are not AHLs again highlighting the evolution away from responding to AHLs of these proteins.

In this mini-review we wish to discuss/review that LuxR solos are evolving away from binding AHLs and can belong to an interkingdom circuit between PAB and plants. In addition very recent results also indicate that LuxR solos have evolved to be part of QS systems that produce and respond to molecules which are not AHLs. From the sequencing of proteobacterial genomes it is evident that LuxR solos are common and widespread meaning that many different forms of signaling could have evolved from the canonical AHL QS systems.

## QS LuxR-FAMILY PROTEINS AND INTERKINGDOM SIGNALING WITH PLANTS

A LuxR solo sub-family has been recently discovered which are only found in PAB that do not bind AHLs but to plant produced compounds (Gonzalez and Venturi, 2013; Venturi and Fuqua, 2013). These LuxRs are very closely associated to QS LuxRs differing in the conservation of one or two of the six highly conserved amino acids in the AHL-binding domain. An AHL QS system widely distributed among PAB has therefore evolved away from being a bacterial intercellular signaling pathway and is now part of an interkingdom circuit between bacteria and plants. Compared to canonical QS LuxRs, this solo sub-family lacks some conservation in the AHL-binding domain which most probably allows them to bind to plant low molecular weight compounds rather than AHLs (Ferluga and Venturi, 2009).

Five members of this subfamily have been studied and these are XccR of *Xanthomonas campestris *pv.* campestris *(*Xcc*), OryR of *Xanthomonas oryzae *pv.* oryzae *(*Xoo*), PsoR of *Pseduomonas fluorescens*, XagR of *Xanthomonas axonopodis *pv.* glycines *(*Xag*)**and NesR of *Sinorhizobium meliloti *(Ferluga et al., 2007; Zhang et al., 2007; Ferluga and Venturi, 2009; Patankar and Gonzalez, 2009; Subramoni et al., 2011; Chatnaparat et al., 2012). OryR of the rice vascular pathogen *Xoo *is involved in virulence, it responds to plant signals and activates the expression of the neighboring *pip* and of motility genes (Ferluga et al., 2007; Ferluga and Venturi, 2009; Gonzalez et al., 2013). XccR of the crucifer pathogen* Xcc* also responds to a yet unidentified plant compound and regulates the neighboring *pip* gene (Zhang et al., 2007). XagR of the soybean pathogen *Xag* which causes bacterial leaf pustule on soybean (*Glycine max*) is also involved in virulence (Chatnaparat et al., 2012). Like XccR in *Xcc*, XagR in *Xag* also activates *pip* transcription *in* *planta* and temporal studies have indicated that *pip *transcription increases gradually after infection, reaching greatest activity after 72 h, before slowly decreasing. This observation could be due to a plant compound(s) which are produced by the plant in response to pathogen attack by *Xag*. Interestingly, a similar observation has been made with *pip* regulation by OryR in *Xoo* (Ferluga and Venturi, 2009). XagR is also involved in the negative regulation of adhesion via *yapH* in response to plant compound(s) facilitating the spread of the pathogen in the plant (Chatnaparat et al., 2012). Two of these LuxR-type proteins have been studied in plant-beneficial bacteria, namely PsoR of *P. fluorescens* and NesR of *S. meliloti *(Patankar and Gonzalez, 2009; Subramoni et al., 2011). PsoR responds to plant compounds of several plant species and plays a role in biocontrol of rhizospheric *P*. *fluorescens* by controlling transcriptional regulation in response to plant compound(s), of various anti-microbial-related genes (Subramoni et al., 2011). NesR of *S. meliloti* has been associated with survival under stress and utilization of various carbon sources (Patankar and Gonzalez, 2009).

Importantly, homologs of OryR/XccR/XagR are also present in the major genera of PAB including *Dickeya, Xanthomonas*, *Agrobacterium, Pseudomonas, Rhodospirillum*, *Citreicella*, *Rhizobium*, * Sinorhizobium* (Gonzalez and Venturi, 2013). Homologs are only present in PAB meaning that this sub-family evolved to respond to plant compounds. This sub-family therefore represents a widespread uni-directional interkingdom signaling between plants and bacteria. Interestingly all genes of this *luxR* family have an adjacent *pip* gene (in some cases two, one on each side of the gene) questioning the possible role of *pip* in this circuit.

## LuxR SOLOS AS PART OF NOVEL QS SYSTEMS

A very recent finding in the insect pathogen *Photorhabdus luminiscens* has opened up new directions toward LuxR responsiveness to bacterial endogenous molecules that are not AHLs. The *P. luminiscens *genome was found to possess two LuxR solos; one was highly homologous to SdiA of *S. enterica* and was therefore predicted to bind AHLs whereas the other LuxR solo, designated PluR, was found to respond to a new class of endogenously produced signaling molecules that are not AHLs. PluR was found detecting α-pyrones which are endogenously produced by a ketosynthase called PpyS. PluR is therefore part of a novel QS system and PpyS/PluR represents a new type of cell-cell communication circuit shown to regulate cell-clumping in *P. luminiscens *(Brachmann et al., 2013). This is another example where LuxR solos have evolved away from the ability to bind to AHLs; it is therefore likely that since many LuxR solos are present in proteobacteria, several will most probably bind and respond to different signal molecules. Interestingly, the majority of PAB have multiple LuxR solos; some of them being related to AHL binding, others binding to plant compounds but some will most probably be involved in binding other signaling molecules, either endogenous or exogenous, which have not yet been identified.

## PERSPECTIVES

**FIGURE 1:**
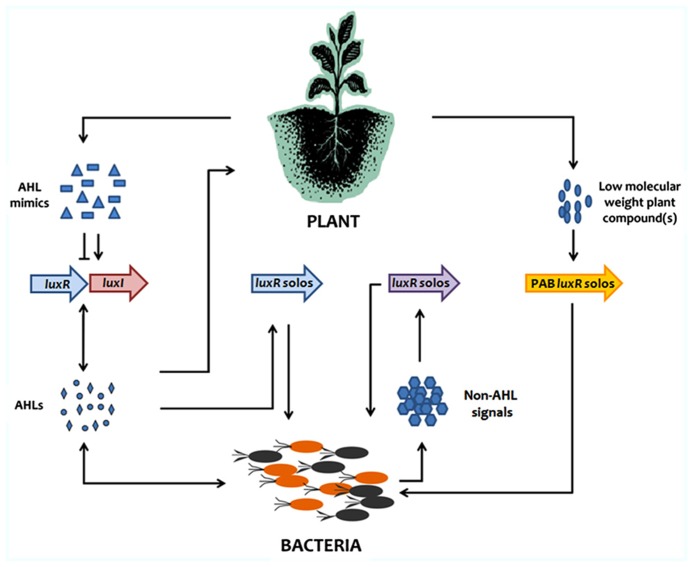
**Summary of current mode of action of AHL QS and of LuxR solos in signaling between plants and bacteria**.

LuxR solos which are closely related to QS AHL LuxRs are widespread among proteobacteria. Recent studies have evidenced that LuxR solos have evolved away from responding to AHLs and bind to other signals (**Figure 1**). A sub-family of PAB LuxR solos are not involved in a QS response but rather respond to plant signals providing information to the bacterium of its arrival/entry in the plant for both pathogenic and beneficial species. Obviously, the outcome of this interkingdom response varies between pathogenic and beneficial bacteria as revealed for example with regulon studies of OryR in *Xoo* and PsoR in *P. fluorescens*. The major step now in better understanding this system is to identify the structure of the plant molecule(s) to which this LuxR-family responds to. This will be a major challenge as plants produce a very large number of low molecular weight secondary metabolites. It is tempting to think that the molecules will be structurally close to AHLs; however, as AHLs do not bind these LuxRs and do not interfere/compete with the plant response it could indicate that they are structurally unrelated. It is possible that different LuxRs of this subfamily bind/respond to different but related plants signals since so many different bacteria possess them interacting with many different plants. We cannot exclude however, that the plant signal is the same being a common compound present in many plants. Recently, the discovery that a LuxR solo responds to a different endogenous signal that is not an AHL opens the way to LuxR solos being part of other types of QS systems. In future work, we need therefore to consider that many LuxR solos may have evolved the ability to respond to a wide variety of signals.

## Conflict of Interest Statement

The authors declare that the research was conducted in the absence of any commercial or financial relationships that could be construed as a potential conflict of interest.
